# POWER ASSIST ADD-ONS FOR OLDER ADULT MANUAL WHEELCHAIR USERS: A SCOPING REVIEW

**DOI:** 10.1093/geroni/igae098.2565

**Published:** 2024-12-31

**Authors:** Oladele Atoyebi, Andrew Wister, Johanne Mattie, Gloria Gutman, Habib Chaudhury, Eireann O’Dea, Sogol Haji Hosseini, Jaimie Borisoff

**Affiliations:** Simon Fraser University, Vancouver, British Columbia, Canada; Simon Fraser University, Vancouver, British Columbia, Canada; British Columbia Institute of Technology, Vancouver, British Columbia, Canada; Simon Fraser University, Vancouver, British Columbia, Canada; Simon Fraser University, Vancouver, British Columbia, Canada; Simon Fraser University, Vancouver, British Columbia, Canada; Simon Fraser University, Vancouver, British Columbia, Canada; British Columbia Institute of Technology, Vancouver, British Columbia, Canada

## Abstract

Manual wheelchairs can promote independence among users of all ages. However, there are age-related differences in the level of disability, strength, stamina, and the environmental conditions within which the wheelchair is used. Using power assist add-ons may mitigate these limitations and help individuals in independent mobility. A gap in the literature is an understanding of the ways in which aging may limit the functionality of power assists on wheelchairs and the impact on independence and active aging. This scoping review analyzes scientific and gray literature to examine the use of power assist add-ons among older adults who use manual wheelchairs, as well as their advantages, limitations, and potential benefits in promoting independence and active aging. This review was guided by the PRISMA checklist for scoping reviews, and the Arksey and O’Malley review methodology. The literature search was carried out in two phases: 1) a keyword and MeSH search of electronic databases, proceedings, as well as symposia for relevant titles/abstracts; and 2) a search of Google and Google Scholar, as well as hand searches. After applying the inclusion and exclusion criteria, we included 20 publications with a focus on power-assist wheelchair technology for full review. Results indicate that power-assist add-ons for manual wheelchairs show promise in improving mobility and reducing user exertion for older adults. However, concerns regarding safety, indoor maneuverability, and user preferences highlight the need for specialized training and retrofitting power assist add-ons, especially among older users. Further research on user-centered design, and adherence to safety standards are warranted.

